# Conjunctival myxoma – atypical presentation of a rare tumour: case report and review of literature

**DOI:** 10.1186/s12886-016-0233-1

**Published:** 2016-05-13

**Authors:** Neharika Sharma, Stephen O’Hagan, Gael Phillips

**Affiliations:** Cairns Base Hospital, 165 The Esplanade, Cairns, Queensland Australia; (Ophthalmology), James Cook University, Queensland, Australia; Pathology Queensland, Royal Brisbane and Women’s Hospital, Queensland, Australia

**Keywords:** Conjunctival myxoma, Conjunctival mass, Conjunctival cyst, Conjunctival tumour, Carney complex

## Abstract

**Background:**

Conjunctival myxomas are rare, benign, connective tissue tumours that classically present as slow-growing, painless, well-circumscribed masses (Arch Ophthalmol 124:735-8, 2006; Case Rep Ophthalmol 3:145-50, 2012). There have been 29 cases reported in the literature (Arch Ophthalmol 124:735-8, 2006; Malays J Med Sci 20(1):92-4, 2013; Case Rep Ophthalmol 3:145-50, 2012; Middle East Afr J Ophthalmol 19(3):353-3, 2012). We present a case with atypical features, and emphasize the importance of excisional biopsies for diagnosing indeterminate conjunctival lesions.

**Case presentation:**

A 32 year old Korean woman presented with a 5 mm × 7 mm × 3 mm pedunculated firm cystic lesion on the inferior palpebral conjunctiva of her right lower eyelid. The lesion had rapidly enlarged over the course of a week. She gave a history of uncomplicated bilateral epiblepharon correction performed in Korea three months prior. There were no systemic features, or family history of genetic conditions. The lesion was excised under local anaesthesia and reported to be a conjunctival myxoma.

The clinical and histopathological features of this lesion were consistent with previous reports on conjunctival myxoma (Arch Ophthalmol 124:735-8, 2006; Arch Ophthalmol 101:1416-20, 1983; Case Rep Ophthalmol 3:145-50, 2012; Am J Ophthalmol 102(1):80-84, 1986). The unusual features of this case were, the rapid growth of the lesion - with the previously documented mean time before presentation being 34 months (range 3 months - 24 years) (Arch Ophthalmol 124:735-8, 2006; Case Rep Ophthalmol 3:145-50, 2012); the location of the lesion in the inferior palpebral conjunctiva - 93 % of previously reported cases had occurred in the bulbar conjunctiva (Arch Ophthalmol 124:735-8, 2006; Case Rep Ophthalmol 3:145-50, 2012); and its occurrence in association with recent eyelid surgery - which has never been reported.

**Conclusion:**

This case of conjunctival myxoma adds to the small number of documented cases, by demonstrating an atypical presentation. Conjunctival myxomas can occur in association with the Carney Complex, which is an autosomal dominant syndrome associated with benign tumours, spotty mucocutaneous pigmentation, and endocrine overactivity (Ophthalmic Surg Lasers Imaging 39(6):514-6, 2008). Ophthalmic manifestations of the Carney Complex have been found to precede vascular embolic events secondary to cardiac myxoma, thus early diagnosis of conjunctival myxoma can prevent potentially devastating consequences (Ophthalmic Surg Lasers Imaging 39(6):514-6, 2008). The different presentations of this rare tumour emphasise the importance of excisional biopsies in diagnosing indeterminate conjunctival lesions; and its association with cardiac myxoma, highlights the need for cardiac investigations in all patients who present with conjunctival myxoma (J Ophthalmol (1);1-5, 2014; Ophthalmic Surg Lasers Imaging 39(6):514-6, 2008).

## Background

Myxomas are benign connective tissue tumours of mesenchymal origin, and can manifest in the heart, skin, bone, skeletal muscle, nasal sinuses, and the gastrointestinal and genitourinary systems [1–5]. Myxomas have been documented in the ocular adnexa, cornea, and conjunctiva [5]. Conjunctival myxomas are a rare occurrence with only 29 previously reported cases in the literature [4, 5]. They are classically described as slow-growing, painless, well circumscribed, yellow-pink, cyst-like masses, with fibrous, vascular, soft tissue trunks [1–3]. We report a case of conjunctival myxoma with atypical features not previously described in the literature.

## Case presentation

### Case report

**Fig. 1:**
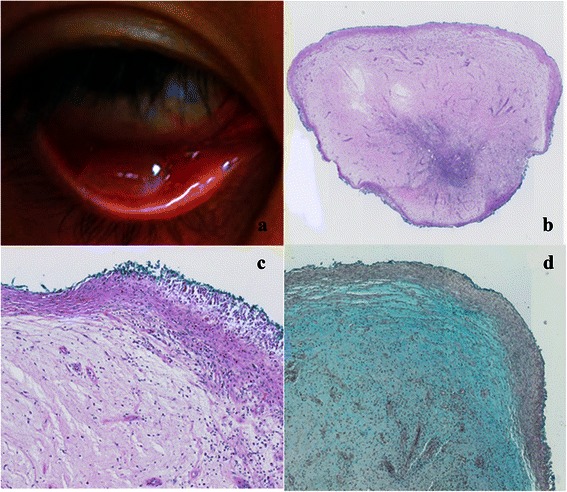
Clinical and histopathological images of conjunctival myxoma*.* **a** 5 mm × 7 mm × 3 mm inferior palpebral conjunctival myxoma OD. **b** Entire lesion at low magnification showing myxoid appearance of the stroma (H&E). **c** Higher magnification showing relatively paucicellular nature of lesion (H&E). **d** Alcian blue stain demonstrating positive reaction for connective tissue mucin in the stroma

A 32 year old Korean woman presented with a one week history of right ocular surface discomfort, and the sudden appearance of a firm cystic lesion fixed to the palpebral conjunctiva of her right lower eyelid by a central trunk (Fig. 1a). The lesion was non-tender, had never ulcerated or haemorrhaged, and was increasing in size daily. At presentation, its dimensions were 5 mm × 7 mm × 3 mm. A history of bilateral epiblepharon correction performed in Korea three months prior was given. The patient was systemically well, and had no other significant ocular or medical history. Her uncorrected visual acuity was 6/5 bilaterally. Extraocular movements were full and painless. Bilateral anterior segment and dilated fundus examinations were unremarkable. The lesion was completely excised at the trunk under local anaesthesia with minimal bleeding. Chloramphenicol ointment QID OD was commenced post-operatively for one week. There were no signs of recurrence one week post-excision. Histopathology demonstrated a central zone of myxoid change (containing spindle-cells, stellate-cells, sparse blood vessels, and patchy inflammation), surrounded by a zone of fibrous connective tissue (Fig. 1b and c). Alcian blue staining was positive for connective tissue mucin (Fig. 1d). Digested Periodic-acid-Schiff (PAS) staining was negative for epithelial mucin. Complete systemic evaluation excluded any further myxomas (including cardiac), unusual areas of pigmentation, and endocrine abnormalities.

### Literature review

Conjunctival myxomas are rare. Grossniklaus et al. (1987) noted four cases of conjunctival myxoma in a review of 2455 conjunctival specimens [4, 6]. Similarly, Shields et al. (2004) reported one case of conjunctival myxoma in a clinical review of 1643 conjunctival lesions [4, 6]. Including this case, the mean age at presentation is 45 years (Table 1) [5–7]. A review of 58 patients with myxomas in other soft tissues found the mean age to be 55 years [2]. No significant trends in gender or racial predisposition have been noted to date, however the number of reported cases remains small (Table 1) [5, 6].

**Table 1:** Clinical findings of the 29 published cases, and this case, of Conjunctival Myxoma

Category		No. of cases (*n* = 30)
Age (mean = 45 years old^a^)	0–20	4
	21–40	7^a^
	41–60	12
	61–80	7
	>81	0
Gender	Male	15
	Female	15^a^
Race	Caucasian	5
	Asian	4^a^
	African	1
	Unrecorded	20
Clinical presentation	Conjunctival Mass	18
	Conjunctival Cyst	9^a^
	Pain & Redness	2
	Disruption to Motility	1
Conjunctival location	Bulbar – Temporal	14
	Bulbar – Nasal	7
	Bulbar – Superior	4
	Bulbar – Not specified	2
	Palpebral – Inferior	2^a^
	Palpebral – Superior	1
Maximum dimension (mean = 10 mm^a^)	0–10 mm	9^a^
	10–20 mm	3
	>20 mm	0
	Unrecorded	18
Time before presentation (mean = 32 months^a^)	<1 month	1^a^
	1 month – 6 months	5
	7 months – 12 months	6
	13 months – 24 months	1
	>24 months	10
	Unrecorded	7
Systemic diseases	Carney Complex	1
	Zollinger-Ellison	1
	Nil associated	28^a^

^a^Indicates/includes this case

Not all articles were available online and findings were obtained from previous literature reviews - Demirci (2006) [2], Chen (2012) [5]

### Review of clinical presentation

Conjunctival myxomas typically present as slow-growing, painless, well circumscribed, yellow-pink, cyst-like masses, with fibrous, vascular, soft tissue trunks [1–3]. Not including this case, the mean timeframe before patients presented for ophthalmic review of their conjunctival myxoma was 34 months (Table 1). This patient’s conjunctival myxoma developed rapidly over one week to a 5 mm × 7 mm × 3mm lesion. Lesions have been reported to range between 4 mm and 20 mm in diameter (Table 1). This lesion reached almost half the recorded maximum size within one week. The majority of cases are painless –although there have been two reported cases of conjunctival myxoma with ocular pain [5]. Ninety percent of reported conjunctival myxomas occurred in the bulbar conjunctiva - with the majority being temporal (Table 1). This is the second case to be documented as arising from the inferior palpebral conjunctiva [8]. There have been no previous reports of conjunctival myxoma developing within close proximity to trauma or ophthalmic surgery [2, 4, 5, 9]. This patient presented with her myxoma within 3 months of having bilateral uncomplicated surgical correction of her congenital epiblepharon - a common occurrence in Asians [10]. We could not determine any connection between her bilateral congenital epiblepharon and conjunctival myxoma. The conjunctival myxoma in this case had a typical clinical appearance. It was atypical because of its rapid growth rate, its unusual location on the inferior palpebral conjunctiva, and its occurrence in association with recent eyelid surgery.

### Review of histology

Histologically, myxomas resemble Wharton’s jelly, the loose mucoid tissue found within the umbilical cord [3, 11]. The characteristic histopathological features of conjunctival myxoma are, sparsely scattered stellate- and spindle-shaped cells distributed throughout a mucinous matrix, with delicate reticulin fibres, minimal blood vessels, and mature collagen fibres [2, 4, 6, 12, 13]. The mucinous matrix is predominantly composed of hyaluronic acid, with a lesser amount of chondroitin sulphate, and has been reported to react to vimentin, Alcian blue, and alpha-smooth-muscle actin staining, suggesting a fibroblastic cell phenotype [4, 6, 7]. It is non-reactive to S-100 protein, desmin, myoglobulin, and digested Periodic-acid-Schiff (PAS) staining [4, 6]. The differential diagnosis of conjunctival myxomas includes, amelanotic naevus, amelanotic melanoma, fibrous histiocytoma, conjunctival cyst, lymphangioma, myxoid neurofibroma, spindle-cell lipoma, rhabdomyosarcoma, and liposarcoma [4–6]. Histologically, an absence of pigmentation, the presence of sparse vascular structures, characteristic cellular morphology, and mucin staining, help differentiate conjunctival myxomas from these lesions [4].

### Review of prognosis

To date all cases of conjunctival myxoma have been treated with excisional biopsy [5, 8]. There have been no previous reports of malignant transformation in a mean follow-up time of 30 months (range 5–132 months) [2, 5, 6]. There has only been one reported case of recurrence, which occurred 12 months after the original excision in a patient with the Carney Complex [6]. In general, the recurrence rate of all myxomas is documented as being relatively low. A review of 58 patients with soft tissue myxomas found a 3 % incidence of recurrence 8–10 months post-excision [2].

### Review of associated systemic diseases

Conjunctival myxoma has been associated with both the Carney Complex and Zollinger-Ellison syndrome [5, 6, 14]. The Carney Complex is an autosomal dominant syndrome that requires at least two of the following criteria for diagnosis: the presence of myxomas; spotty mucocutaneous pigmentation (face, trunk, lips, eyelid, conjunctiva); endocrine overactivity (Cushing’s syndrome, pituitary adenoma, and/or sexual precocity); or psammomatous melanotic schwannomas [6, 14, 15]. Ophthalmic manifestations of the Carney Complex include eyelid lentigines, conjunctival or caruncle spotty pigmentation, and eyelid or conjunctival myxomas [6, 15]. The one case of conjunctival myxoma associated with the Carney Complex exhibited palpebral conjunctival, eyelid, coetaneous, and left ventricular myxomas [6]. Carney reported that greater than 50 % of patients with the Carney Complex suffered a significant embolic event in their lifetime related to cardiac myxomas [15]. Ophthalmic manifestations of the Carney Complex, not limited to myxoma, have been shown to precede embolic events [14, 15]. Early identification of ocular myxomas and subsequent screening and monitoring for cardiac myxoma is recommended.

Conjunctival myxoma has been associated with a case of pancreatic gastrinoma in Zollinger-Ellison syndrome [14]. Zollinger-Ellison syndrome may be a manifestation of the Carney Complex, given the neural crest origins of myxomas, schwannomas (seen in the Carney Complex), and gastrinomas [14]. Conjunctival myxomas have not been reported with other systemic diseases associated with myxomas, such as Mazabraud syndrome (intramuscular myxoma) and McCune-Albright syndrome (intramuscular and coetaneous) [4, 5].

## Conclusion

Conjunctival myxomas are benign tumours, however, they can mimic more sinister lesions, such as amelanotic melanomas. The clinical appearance and histopathological features in this case are consistent with the literature. However, its location and rapid development in close proximity to lower eyelid surgery, initially suggested an inflammatory or infective aetiology. The different presentations of this rare tumour emphasise the importance of excisional biopsy in identifying the pathology of indeterminate conjunctival lesions. Their association with systemic syndromes can lead to the detection of previously undiagnosed cardiac myxomas, which if left undetected, may result in vascular embolic events [14, 15]. We would recommend cardiac screening in all patients diagnosed with a conjunctival myxoma.

### Ethics approval and consent to participate

Not applicable.

### Consent for publication

Written informed consent was obtained from the patient for publication of this case report and any accompanying images, providing no identifying features are released.

### Availability of data and materials

All data supporting our findings will be shared upon request, although the majority is contained within the manuscript.

## Abbreviation

PAS: periodic-acid-Schiff (lines 87, 126)
